# Prevalence, awareness, treatment, and control of hypertension in a small northern town in Nicaragua: The Elieth‐HIFARI study

**DOI:** 10.1002/hsr2.120

**Published:** 2019-04-11

**Authors:** Marion Jose Valladares, Noel A. Rodríguez Sándigo, Ginner O. Rizo Rivera, Marco A. Rodríguez Jarquín, Rosse M. Rivera Castillo, Indiana M. López Bonilla

**Affiliations:** ^1^ Faculty of Medical Sciences UNAN‐Leon León Nicaragua; ^2^ ELAM Cuba Victoria Motta Hospital Jinotega Nicaragua; ^3^ Medicine Faculty UNAN‐Managua Managua Nicaragua; ^4^ Medicine Faculty UCATSE Estelí Nicaragua; ^5^ Research Centre on Health, Work and Environment UNAN‐Leon León Nicaragua

**Keywords:** cardiovascular disease, control rate, high blood pressure, hypertension, prevalence

## Abstract

**Background and aims:**

Hypertension is considered the most important risk factor for cardiovascular disease and is associated with high levels of morbidity, mortality, and health care expenditure. The negative effects of hypertension and its complications are preventable if those at risk are appropriately treated and controlled. Continually monitoring the epidemiological trends of hypertension is essential to formulate and evaluate public health measures to limit its negative effects. The herein presented Elieth‐HIFARI study sought to estimate the prevalence of hypertension, as well as the prevalence of related awareness, treatment, and control in a small town in Central America.

**Methods:**

A population survey to assess cardiovascular risk was conducted (n = 577, 55.3% women, mean age 42.4 years) in the municipality of San Rafael del Norte in northern Nicaragua, between November 2016 and March 2017, based on the STEPwise method by the World Health Organization and the recommendations by the World Hypertension League.

**Results:**

The overall prevalence of hypertension, awareness, treatment, and control was 28.1%, 72.2%, 68.5%, and 36.4%, respectively. Men had a lower prevalence of all indicators (22.5%, 60.3%, 53.4%, and 24.1%, respectively) compared with women (32.6%, 78.8%, 76.9%, and 43.3%, respectively). The median systolic blood pressure was 118.5 mm Hg (20.5 interquartile range [IQR]) (men: 123.0 mm Hg vs women: 115.5 mm Hg, Mann‐Whitney U test *P* < .001), and the mean diastolic blood pressure was 78.0 mm Hg (13 IQR) (men: 77.0, women: 78.0).

**Conclusion:**

Hypertension is highly prevalent in San Rafael del Norte, while control rates are low despite the relatively higher levels of awareness and treatment. Furthermore, women have much higher prevalence of hypertension than men, along with higher awareness, treatment, and control. However, the control rate for those treated for hypertension was low, irrespective of sex.

## 1 INTRODUCTION

Cardiovascular disease (CVD) is the most important cause of mortality worldwide, producing nearly one‐third of all deaths, half of which are premature (defined by the World Health Organization [WHO] as 70 years or younger).^1^, ^2^ Of the 17 million deaths that occur globally each year due to CVDs, 15 million are caused by ischemic heart disease and stroke alone,^1^ with hypertension (HTN) as the most important risk factor for these two major diseases. HTN is also associated with nearly half of the deaths related to ischemic heart disease and stroke,^3^ close to 9.4 million annually.^4^ The high mortality, disability, and social and economic impact of CVDs generate an important burden on health systems and on the productivity of society in general.^4^, ^5^ Specifically, HTN alone has been linked to at least half of the economic loss due to noncommunicable diseases, roughly 2% of the gross domestic product (GDP) of middle‐ and low‐income countries.^5^

Nicaragua has the highest CVD mortality rate in Central America.^2^, ^6^ Managua, the capital of Nicaragua, has the highest prevalence of HTN among various others Central‐American capitals.^7^ In San Rafael del Norte, a small town in northern Nicaragua, HTN‐related visits are among the three most important reasons for seeking medical attention and constitute nearly a third of medical visits for the elderly.^8^ CVDs are also the first causes of mortality there, causing more than 25% of all deaths in those 14 years old and older.^9^

Prevention of HTN‐related complications is a challenge because HTN itself rarely causes symptoms, and therefore, it is usually underdiagnosed, undertreated, and not controlled properly. This is one of the reasons why the WHO considers its early detection and adequate management to be fundamental in order to reduce the risk of complications and death.^3^ Because of this, it is necessary to know the prevalence of HTN and other indicators such as prevalence of awareness, treatment, and control, as proposed by the World Hypertension League (WHL).^10^ These indicators are also essential to identify populations at higher risk for cardiovascular complications, help characterize these groups, and aid in the formulation of prevention and control strategies. Furthermore, they also help to assess where to focus these efforts and monitor and evaluate the progress made by public health policies.^10^, ^11^

Research on HTN prevalence, awareness, treatment, and control has been conducted in many regions of the world, with varying results. Prevalence of HTN has been reported to be as low as 13.4% in Bogota^12^ up to as high as 42.5% in several cities of Chile, Argentina, and Uruguay.^13^ In comparison, many other regions of the world have a prevalence that falls within this range, such as Canada (20%),^14^ China (29.6%),^15^ Korea (33.7%),^16^ Lebanon (36.9%),^17^ and Estonia (36%).^18^ Further variability is seen even within regions and countries, seemingly due to different age compositions of the studied population as the main reason, as well as differences in resources, education, and access to care.

Awareness, treatment, and control rates have also been found to vary widely. Awareness has been measured from as low as 24.6% in a Korean study,^16^ up to as high as 74% in Canada^14^ and 80% in Estonia.^18^ Overall, most studies report that at least more than half of the studied population have awareness.^12^, ^13^, ^14^, ^17^, ^18^ Pharmacologic treatment rates among those hypertensive have been observed ranging from 34.1% in China^15^ up to 60.5% in Italy^19^ and 78.6% in Korea.^16^ However, despite awareness and treatment rates, overall control rates have been found to be consistently low (less than 30%) in comparison, as seen in countries like Italy,^19^ China,^15^ Korea,^16^ and Lebanon,^17^ and in Latin American cities such as Bogota, Buenos Aires, Lima, and Santiago.^12^ Additionally, there are reports of control rates among those treated with medication of between 27.4% (China)^15^ and 72.0% (Canada).^14^

Within the numerous studies on HTN prevalence and associated indicators referred to previously, generally, men have higher prevalence, and women have higher awareness, treatment, and control rates. In addition, older ages (65 years or older) have been reported to have higher prevalence of HTN^12^; however, the association, if any, between age and awareness, treatment, and control is unclear.

Considering the recent publication of the aforementioned standards for reporting HTN by the WHL, and the scarce data related to its core indicators in Nicaragua and Central America, the Elieth‐HIFARI study sought to provide initial reference data for the epidemiological surveillance of such prevalence indicators in a typical small town of northern Nicaragua, considered as such based on shared social, economic, cultural, topographical, and climatological traits.

## 2 METHODS

### 2.1 Study population

A population‐based cross‐sectional survey was conducted in the small town of San Rafael del Norte, in the northern mountainous region of Nicaragua. A sample size of 636 participants was estimated considering an expected HTN prevalence of 25%, based on the most recent prevalence reported for Managua,^7^ with a 95% confidence interval (CI), 5% precision margin, and a 10% expected nonresponse rate. This sample size represents an 18.3% of the total adult population, of 3477 individuals. A random stratified sampling method was employed accounting for sex and age groups, based on the official population census of 2010 to 2016 of the municipality. Participants were selected randomly from a community census provided by the local offices of the national health ministry (MINSA). Persons 18 years of age or older were included, except women who were pregnant or had given birth less than 40 days prior to the interview; persons with any mental or physical disability that impeded the interview or measurement taking were also excluded.

### 2.2 Data collection

The survey was conducted on the basis of door‐to‐door visits to each of the selected participants' residence where the interview took place, after they were given a detailed explanation of the nature of the visit. After agreeing to the interview, participants were asked to sign an informed consent form in order to participate in the study. If at the time of the visit the participant did not meet the ideal conditions for the unbiased measurement of blood pressure (BP) as stated in the Recommendations for the Measurement of Blood Pressure in Humans by the American Heart Association,^20^ then another time for the interview was agreed upon. The team of interviewers, consisting of medical students, underwent previous training to standardize questioning and procedure methods in order to minimize observer bias.

The instrument used for data collection was based upon the first two steps of the Pan American version of STEPS Instrument 3.1 of the “WHO STEPwise approach to non‐communicable disease risk factor surveillance (STEPS)”.^21^ The first step regarded sociodemographic characteristics, history of HTN diagnosis, and medication use for HTN; the second step consisted of physical measurements. The sex of each participant was determined by the interviewer, and ethnicity was reported by the participant from a list of possible options provided by the local Health Ministry's Census (ie, Mestizo, White, Black, Chorotega, Miskitu, Sumu‐Mayagna, and Other). BP was measured at the end of each interview and in accordance with the guidelines of the American Heart Association^20^ and the STEPS Manual.^22^ Participants were instructed to remain still in a seated position for 15 minutes without crossing their legs. Afterwards, the arm cuff was placed on the left arm 2 cm above the elbow crease with the palm facing up and rested on a support at the heart level. Three readings of BP were taken 3 minutes apart, and the mean of the last two readings was used for analysis. The automatic digital BP monitor used was the Omron M6 Comfort model HEM‐7321‐E (Kyoto, Japan), validated by the protocols of the European Society of Hypertension.^23^ Analysis of the risk factors for HTN and anthropometry values collected in this study will be reported in future publications.

### 2.3 Ethical considerations

The present study was done in keeping with the Declaration of Helsinki (2013), and it was remitted to and approved by the Medical Ethics Committee of the National Autonomous University of Nicaragua in Leon. At the end of each interview, participants were informed of their results and received pertinent prevention and health promotion counseling; those who had high BP readings were remitted to appropriate health care services depending on their severity.

### 2.4 Definitions

We used the definitions recommended by the WHL for core indicators and analysis for HTN surveillance.^10^ Prevalence of HTN was defined as the proportion of participants who had a systolic blood pressure (SBP) ≥ 140 mm Hg or a diastolic blood pressure (DBP) ≥ 90 mm Hg, or who reported currently taking medication for high BP for at least the last 2 weeks. Prevalence of awareness was defined as the proportion of participants with HTN who took medication for high BP or were diagnosed with HTN by a health professional; treatment, as the proportion of participants with HTN who took medication for high BP; and controlled HTN, as the proportion of participants with HTN who took medication for high BP and had SBP < 140 mm Hg and DBP < 90 mm Hg. Prevalence of “treatment among those aware” was defined as the proportion of participants who took medication for HTN among those aware and “control among those treated”, as the proportion of participants who had a SBP < 140 mm Hg and DBP < 90 mm Hg among those treated. Prevalence of optimal BP was defined as SBP < 120 mm Hg and DBP < 80 mm Hg in the absence of treatment for HTN; pre‐hypertension (Pre‐HTN), as the proportion of participants having SBP > 120 mm Hg and < 140 mm Hg and/or DBP > 80 mm Hg and < 90 mm Hg, without having HTN; and isolated systolic HTN, as the proportion of participants with HTN who had SBP ≥ 140 mm Hg and DBP < 90 mm Hg.

### 2.5 Statistical analysis

The independent variables studied included age, sex, ethnicity, marital status, education, occupation, income level, SBP, DBP, previous diagnosis of HTN, and medication use for high BP. The sociodemographic variables of the population were summarized and reported with corresponding prevalence rates and 95% CIs. Four age groups were defined as recommended by the WHO, and income level was categorized in quartile groups based on household income per capita. The Median SBP and DBP as well as the interquartile range, are reported for sex and age groups. Likewise, the prevalence of HTN, awareness, treatment and control, treatment among aware, control among treated, optimal BP, Pre‐HTN, and isolated systolic HTN were all estimated for sex and age groups, with 95% CIs.

The median values of SBP and DBP were compared by sex groups using the Mann‐Whitney U test and age groups using the Kruskal‐Wallis H test. The data were stratified by sex, as is recommended by the WHL^10^ and the WHO.^22^ The prevalence of the indicators mentioned above was compared by sex, age groups, and selected demographic characteristics using the Pearson chi‐squared test. A two‐sided *P* value < .05 was considered statistically significant. All data were introduced and processed using the IBM SPSS version 22 statistical software.

Normality tests were conducted for age, household income per capita, mean SBP, and mean DBP. None of the tested variables had a normal distribution (per Shapiro‐Wilk test, *P* < .05), observing that while histograms for both SBP and DBP closely resembled a normal distribution, they were slightly skewed to the right.

## 3 RESULTS

### 3.1 Characterization of the population

From November 2016 to January 2017, a total of 577 participants were enrolled in the Elieth‐HIFARI study, out of the estimated sample size of 636 adults. The median age was 40 years (25 IQR), and 55.3% were women. More than 91% of participants self‐identified as Mestizo (or mixed ethnicity). Nearly half of the population had only a primary level of education or less, and there was a literacy rate of 95.5%. Unemployment was at a 4.1%, and the main occupations were housemaker in women and self‐employed in men; 8.7% of the population were students. The majority of participants lived with a life partner; 53.9% were either married or cohabitating. The median household income per capita per month was US $56.60 (in January 2017)^24^: less than $3 a day. All four income level groups that are based on quartiles (Q1 = $33.96, Q3 = $101.88) had similar proportions. Besides occupation (*P* < .001, per Mann‐Whitney U test), no significant differences were found between men and women in other demographic variables. Table 1 summarizes the selected sociodemographic characteristics of the participants.

**Table 1:** Distribution of sociodemographic characteristics of the population of San Rafael del Norte (n = 577)

	No.	Percentage	95% CI
Age			
18‐29	153	26.5	23.1‐30.3
30‐49	223	38.6	34.7‐42.8
50‐69	165	28.6	25.0‐32.1
70+	36	6.2	4.3‐8.3
Sex			
Male	258	44.7	40.6‐49.0
Female	319	55.3	51.0‐59.4
Ethnicity			
Mestizo	527	91.3	88.7‐93.4
Blanco (White)	40	6.9	4.9‐9.2
Negro (Black)	10	1.7	0.7‐2.9
Education			
<Primary	100	17.3	14.0‐20.3
Primary	187	32.4	28.6‐36.4
Secondary	171	29.6	26.0‐33.4
College and +	119	20.6	17.3‐24.1
Occupation			
Government employee	78	13.5	10.9‐16.5
Nongovernment employee	110	19.1	15.9‐22.4
Self‐employed	142	24.6	21.3‐28.6
Student	50	8.7	6.4‐11.1
Homemaker, other^a^	155	26.9	22.9‐30.7
Retired	18	3.1	1.7‐4.7
Unemployed	24	4.2	2.6‐5.9
Marital status			
Single	227	39.3	35.5‐43.3
With life partner	311	53.9	49.9‐57.9
Separated/divorced	17	2.9	1.7‐4.3
Widowed	22	3.8	2.3‐5.4
Income level (n = 429)			
Low	113	26.3	22.13‐30.47
Lower middle	109	25.4	21.28‐29.52
Higher middle	93	21.7	17.80‐25.6
High	114	26.6	22.42‐30.78

a Nonpaid.

### 3.2 Distributions of SBP and DBP

The overall median SBP was 118.5 mm Hg (20.5 IQR), and median DBP was 78.0 mm Hg (13.0 IQR). Overall, men had a higher median SBP than women (123.0 vs 115.5 mm Hg, respectively, *P* < .001, Mann‐Whitney U test); however, no significant differences were observed based on sex in older age groups (50‐69 years, men: 128.8 mm Hg vs women: 122.5 mm Hg, *P* = .083, Mann‐Whitney U test; 70+ years, men: 135.3 mm Hg vs women: 138.0 mm Hg, *P* = .628, Mann‐Whitney U test). We observed no overall difference in the median DBP between men and women (77.0 vs 78.0 mm Hg respectively, *P* = .276, Mann‐Whitney test). The median SBP increased with age (18‐29 years: 111.5 mm Hg, 30‐49 years: 117.0 mm Hg, 50‐69 years: 124.0 mm Hg, 70+: 135.0 mm Hg, *P* < .001, Kruskal‐Wallis H test), as did the median DBP (18‐29 years: 75.0 mm Hg, 30‐49 years: 78.0 mm Hg, 50‐69 years: 81.0 mm Hg, 70+: 78.0 mm Hg, *P* < .001, Kruskal‐Wallis H test), but only up to 50 years, after which no differences were observed (*P* = .125, Mann‐Whitney U test). Table 2 summarizes the distribution of SBP and DBP values by sex and age groups.

**Table 2:** Median and IQR of SBP and DBP

	Both sexes			Men			Women		
	No.	Median	IQR	No.	Median	IQR	No.	Median	IQR
SBP, mm Hg									
Overall (crude)	577	118.50	20.50	258	123.00	19.50	319	115.50	18.00
Age group, y									
18‐29	153	111.50	19.50	74	119.50	19.50	79	106.00	16.00
30‐49	223	117.00	16.00	100	119.50	12.50	123	114.00	15.00
50‐69	165	124.00	24.50	66	128.75	23.00	99	122.5	23.00
70+	36	135.00	22.00	18	135.25	27.00	18	138.00	23.00
DBP, mm Hg									
Overall (crude)	577	78.00	13.00	258	77.00	13.00	319	78.00	13.00
Age group									
18‐29	153	75.00	11.50	74	75.00	14.00	79	75.00	10.50
30‐49	223	78.00	12.50	100	76.25	13.75	123	78.00	10.50
50‐69	165	81.00	13.00	66	81.50	12.00	99	81.00	12.50
70+	36	78.00	13.75	18	76.50	8.50	18	79.25	18.50

Abbreviations: DBP, diastolic blood pressure; IQR, interquartile range; SBP, systolic blood pressure.

### 3.3 Prevalence, awareness, treatment, and control of HTN

The crude prevalence of HTN found in our population was 28.1% (95% CI, 24.6‐32.1), which was 10% higher in women compared with men (32.6% [95% CI, 27.3‐37.9] vs 22.5% [95% CI, 17.1‐27.9]), and increased significantly in older age groups (18‐29 years: 4.6% [95% CI, 1.3‐8.5]; 30‐49 years: 19.3% [95% CI, 13.9‐24.2]; 50‐69 years: 52.7% [95% CI, 44.8‐60.6]; 70+ years: 69.4% [95% CI, 52.8‐83.3]). Additionally, HTN prevalence was also statistically higher in those with a college level, primary, or less than primary education than in participants with secondary education only, higher in the retired, and lowest in students (see Tables 3 and 4). Other occupation categories had similar prevalence. Widows had the highest HTN prevalence, singles had the lowest, and those married or cohabitating had similar prevalence to those divorced or separated. No significant differences in HTN prevalence were found by income levels. Table 3 shows prevalence rates for each indicator by sex and age, and Table 4 shows prevalence rates for other sociodemographic variables and comparisons by each variable.

**Table 3:** Hypertension prevalence, awareness, treatment, control, treatment among aware, and control among treated

	Prevalence		Awareness		Treatment		Control		Treatment among aware		Control among treated	
	No. (%)	95% CI	No. (%)	95% CI	No. (%)	95% CI	No. (%)	95% CI	No. (%)	95% CI	No. (%)	95% CI
Both sexes												
Overall (crude)	162 (28.1)	24.6‐32.1	117 (72.2)	65.4‐79.0	111 (68.5)	61.1‐75.9	59 (36.4)	29.0‐43.8	111 (94.9)	90.6‐98.3	59 (53.2)	44.1‐62.2
Age group												
18‐29	7 (4.6)	1.3‐8.5	0 (0.0)	‐	0 (0.0)	‐	0 (0.0)	‐	0 (0.0)	‐	0 (0.0)	‐
30‐49	43 (19.3)	13.9‐24.2	28 (65.1)	51.2‐79.1	25 (58.1)	44.2‐74.4	11 (25.6)	14.0‐39.5	25 (89.3)	75.1‐100	11 (44.0)	24.0‐64.0
50‐69	87 (52.7)	44.8‐60.6	70 (80.5)	71.3‐88.5	69 (79.3)	70.1‐87.4	38 (43.7)	33.3‐54.0	69 (98.6)	95.7‐100	38 (55.1)	43.5‐66.7
70+	25 (69.4)	52.8‐83.3	19 (76.0)	56.0‐92.0	17 (68.0)	48.0‐84.0	10 (40.0)	20.0‐60.0	17 (89.5)	73.7‐100	10 (58.8)	35.3‐82.4
Men												
Overall (crude)	58 (22.5)	17.1‐27.9	35 (60.3)	48.3‐72.4	31 (53.4)	39.7‐65.5	14 (24.1)	13.8‐36.2	31 (88.6)	77.1‐97.1	14 (45.2)	29.0‐64.5
Age group												
18‐29	4 (5.4)	1.4‐10.8	0 (0.0)	‐	0 (0.0)	‐	0 (0.0)	‐	0 (0.0)	‐	0 (0.0)	‐
30‐49	15 (15.0)	8.0‐22.0	10 (66.7)	40.0‐86.7	9 (60.0)	33.5‐80.0	2 (13.3)	0.0‐33.3	9 (90.0)	70.0‐100	2 (22.2)	0.0‐55.3
50‐69	28 (42.4)	31.8‐56.0	16 (57.1)	39.3‐75.0	15 (53.6)	35.7‐71.4	7 (25.0)	10.7‐42.9	15 (93.8)	81.3‐100	7 (46.7)	20.0‐73.3
70+	11 (61.1)	38.9‐83.3	9 (81.8)	54.5‐100	7 (63.6)	36.4‐90.9	5 (45.5)	18.2‐72.7	7 (77.8)	44.4‐100	5 (71.4)	42.9‐100
Women												
Overall (crude)	104 (32.6)	27.3‐37.9	82 (78.8)	71.2‐86.5	80 (76.9)	69.2‐84.6	45 (43.3)	33.7‐52.9	80 (97.6)	93.9‐100	45 (56.3)	45.0‐67.5
Age group												
18‐29	3 (3.8)	0.0‐7.6	0 (0.0)	‐	0 (0.0)	‐	0 (0.0)	‐	0 (0.0)	‐	0 (0.0)	‐
30‐49	28 (22.8)	16.3‐30.9	18 (64.3)	46.4‐82.1	16 (57.1)	39.3‐75.0	9 (32.1)	14.3‐50.0	16 (88.9)	72.0‐100	9 (56.3)	31.3‐81.3
50‐69	59 (59.6)	50.5‐68.7	54 (91.5)	83.1‐98.3	54 (91.5)	83.1‐98.3	31 (52.5)	39.0‐66.1	54 (100)	‐	31 (57.4)	44.5‐70.4
70+	14 (77.8)	55.6‐94.4	10 (71.4)	42.9‐92.9	10 (71.4)	42.9‐92.9	5 (35.7)	14.3‐64.3	10 (100)	‐	5 (50.0)	20.0‐80.0

Abbreviation: CI, confidence interval.

**Table 4:** Hypertension prevalence, awareness, treatment, and control by demographic characteristics

	No.	Prevalence, %	Awareness, %	Treatment, %	Control, %	Treatment a/aware	Control a/treated
Sex		*P* = .007	*P* = .012	*P* = .002	*P* = .015	*P* = .056^a^	*P* = .294
Male	258	22.5	60.3	53.4	24.1	88.6	45.2
Female	319	32.6	78.8	76.9	43.3	97.6	56.3
Age groups		*P* < .001	*P* < .001	*P* < .001^a^	*P* = .013^a^	*P* = .082^a^	*P* = .559^a^
18‐29	153	4.6	0.0	0.0	0.0	0.0	0.0
30‐49	223	19.3	65.1	58.1	25.6	89.3	44.0
50‐69	165	52.7	80.5	79.3	43.7	98.6	55.1
70+	36	69.4	76.0	68.0	40.0	89.5	58.8
Education		*P* < .001	*P* = .236	*P* = .079	*P* = .059	*P* = .191^a^	*P* = .227
<Primary	100	34.0	82.4	79.4	41.2	96.4	51.9
Primary	187	32.6	75.4	73.8	44.3	97.8	60.0
Secondary	171	15.8	63.0	51.9	14.8	82.4	28.0
College and +	119	33.6	65.0	62.5	35.0	96.2	56.0
Occupation		*P* < .001	*P* = .002^a^	*P* = .005^a^	*P* = .018^a^	*P* = .275^a^	*P* = .282^a^
Government employee	78	28.2	59.1	59.1	31.8	100	53.8
Nongovernment	110	19.1	52.4	52.4	28.6	100	54.5
Self‐employed	142	28.2	62.5	55.0	20.0	88.0	36.4
Student	50	2.0	0.0	0.0	0.0	0.0	0.0
Homemaker and other^b^	155	36.1	85.7	82.1	50.0	95.8	60.9
Retired	18	72.2	92.3	92.3	61.5	100	66.7
Unemployed	24	37.5	88.9	77.8	22.2	87.5	28.6
Marital status		*P* < .001	*P* = .081	*P* = .159^a^	*P* = .177^a^	*P* = .438^a^	*P* = .480^a^
Single	227	18.5	57.1	54.8	23.8	95.8	43.5
With life partner	311	30.2	76.6	72.3	39.4	94.4	54.4
Separated^c^	17	41.2	85.7	71.4	57.1	83.3	80.0
Widowed	22	86.4	78.9	78.9	42.1	100	53.3
Income level		*P* = .653	*P* = .312	*P* = .302	*P* = .675	*P* = .496^a^	*P* = .807
Low	113	24.8	82.1	82.1	46.4	100	56.5
Lower middle	109	25.7	64.3	64.3	39.3	100	61.1
Higher middle	93	23.7	59.1	59.1	36.4	100	61.5
High	114	30.7	68.6	65.7	31.4	95.8	47.8

*Note*. *P* value derived from Pearson chi‐squared test, unless otherwise stated.

a Likelihood ratio *P* value.

b Nonpaid

c Divorced.

The crude prevalence of awareness was 72.2% (95% CI, 65.4‐79.0), which was higher in women than in men, and 0% in participants younger than 30 years. In older age groups, no significant differences were found, neither by education level, marital status, or income level. Considering occupation, homemakers, the retired, and the unemployed had higher awareness, whereas students had 0%. Table 4 summarizes HTN prevalence, awareness, treatment, and control by demographic characteristics.

A total of 68.5% (95% CI, 61.1‐79.5) of participants with HTN were taking medication to lower BP. Higher treatment rates were found in women, older age groups, the retired, homemakers, and the unemployed. No differences were found by education, marital status, or income levels. Treatment rate among participants who were aware of having HTN was at 94.9% (95% CI, 90.6‐98.3); no differences were found by sociodemographic categories in this regard (see Table 4).

The overall control rate was 36.4% (95% CI, 29.0‐43.8), and in those who were taking treatment, it was 53.2% (95% CI, 44.1‐62.2). Overall control was higher in women, older age groups, the retired, and homemakers. Additionally, when considering only those treated for HTN, no differences in control rates were found by any sociodemographic characteristic (see Table 4).

### 3.4 BP categories

Overall, 40.6% (95% CI, 36.7‐44.9) of our population had optimal BP, which was higher in women (44.2% [95% CI, 38.6‐50.2] vs men: 36.0% [95% CI, 29.8‐41.9]) and in younger age groups (18‐29 years: 59.5% [95% CI, 51.6‐67.3] vs 70+ years: 11.1% [95% CI, 2.8‐22.2]). However, when compared by sex and age groups, only women between 18 and 29 years had higher prevalence than men of the same age. In contrast, the prevalence of Pre‐HTN was higher in men than women (41.5% [95% CI, 35.3‐47.7) vs 23.2% [95% CI, 18.5‐27.9]). The overall prevalence of Pre‐HTN was 31.4% (95% CI, 27.4‐35.2), with no significant differences by age group. When compared by sex and age groups, men aged 18 to 29 and 30 to 49 had higher prevalence of Pre‐HTN than their female counterparts, but no difference between them was observed in older age groups. The overall prevalence of isolated systolic HTN was 6.1% (95% CI, 4.3‐7.8); no differences between sexes were observed. However, the rate increased in older age groups, mainly in those 70 and older. Table 5 summarizes the prevalence of optimal BP, Pre‐HTN, HTN, and isolated systolic HTN in the overall population, in each sex and age group. The prevalence of each category is also compared between sexes and age groups separately, as well as between each corresponding age group of males and females.

**Table 5:** Prevalence of blood pressure categories by sex and age groups

	No.	Optimal BP, % (95% CI)	Pre‐HTN, % (95% CI)	HTN, %, (95% CI)	Isolated systolic HTN, % (95% CI)
Both sexes		*P* = .047	*P* < .001	*P* = .007	*P* = .902
Overall (crude)	577	40.6 (36.7‐44.9)	31.4 (27.4‐35.2)	28.1 (24.4‐31.5)	6.1 (4.3‐7.8)
Age group		*P* < .001	*P* = .107	*P* < .001	*P* < .001
18‐29	153	59.5 (51.6‐67.3)	35.9 (28.8‐43.8)	4.6 (1.3‐8.5)	0.7 (0.0‐2.0)
30‐49	223	47.1 (40.8‐53.4)	33.6 (27.4‐40.4)	19.3 (14.3‐24.2)	0.9 (0.0‐2.2)
50‐69	165	20.6 (14.5‐26.7)	26.7 (20.0‐33.3)	52.7 (45.0‐60.0)	12.7 (7.9‐18.2)
70+	36	11.1 (2.8‐22.2)	19.4 (8.3‐33.3)	69.4 (55.6‐83.3)	30.6 (16.7‐47.2)
Men					
Overall (crude)	258	36.0 (29.8‐41.9)	41.5 (35.3‐47.7)	22.5 (17.8‐27.9)	6.2 (3.5‐9.3)
Age group					
18‐29	74	43.2 (31.1‐55.4)*	51.4 (40.5‐63.5)*	5.4 (1.4‐10.8)	1.4 (0.0‐4.1)
30‐49	100	43.0 (34.0‐53.0)	42.0 (33.0‐51.0)*	15.0 (9.0‐23.0)	2.0 (0.0‐5.0)
50‐69	66	24.2 (13.7‐34.8)	33.3 (21.2‐45.5)	42.4 (30.3‐54.5)*	13.6 (6.1‐22.7)
70+	18	11.1 (0.0‐27.8)	27.8 (11.1‐50.0)	61.1 (38.9‐83.3)	22.2 (5.6‐38.9)
Women					
Overall (crude)	319	44.2 (38.6‐50.2)	23.2 (18.5‐27.9)	32.6 (27.6‐37.9)	6.0 (3.4‐8.8)
Age group					
18‐29	79	74.7 (64.6‐83.5)	21.5 (12.7‐31.6)	3.8 (0.0‐8.9)	0.0 (0.0‐0.0)
30‐49	123	50.4 (41.5‐59.3)	26.8 (19.5‐35.0)	22.8 (15.4‐30.9)	0.0 (0.0‐0.0)
50‐69	99	18.2 (11.1‐25.3)	22.2 (15.2‐30.3)	59.6 (50.5‐68.7)	12.1 (6.1‐19.2)
70+	18	11.1 (0.0‐27.8)	11.1 (0.0‐27.8)	77.8 (55.6‐94.4)	38.9 (16.7‐61.1)

*Note*. The prevalence of each category was compared both between sexes and among age groups, as well as between each corresponding age group of males and females. *P* values were derived from Pearson chi‐squared test.

Abbreviations: BP, blood pressure; HTN, hypertension; Pre‐HTN, pre‐hypertension.

* Statistically significant (*P* < 0.05) difference between men and women in the prevalence in the corresponding age group.

## 4 DISCUSSION

This study aimed to provide a starting point in measuring the prevalence, awareness, (pharmacologic) treatment, and control rates of HTN in a typical small town of Nicaragua. We found that HTN is a significant health problem in this population, where 28.1% met the criteria for diagnosis, 31.4% had Pre‐HTN, and only 40.6% had optimal BP. Once adjusted for age and sex to the national population of Nicaragua, our HTN prevalence was 24.3%, compared with an adjusted 28.9% for Managua from a crude 24.8% reported by the Pan American Health Organization in its 2010 Central American Diabetes Initiative (CAMDI) survey.^25^ Even though the comparison of prevalence rates between other studies is difficult given the different sampling methods, inclusion criteria, and population distribution by age and sex, the crude prevalence of HTN in the Elieth‐HIFARI study is comparable with other Latin American cities with higher HTN prevalence such as Buenos Aires, Santiago, and Barquisimeto, all between 20% and 30%,^12^ but higher than that in cities like Lima (12.6%), Bogotá (13.4%), Quito (8.6%), and Mexico City (11.7%).^12^ The rates observed are lower than national estimates of Asian countries like China (29.6%)^15^ and Korea (33.7%).^16^

The majority of participants with HTN were aware and had treatment (72.2% and 68.5%, respectively), and almost all of those aware had treatment (94.9%); however, despite this, the control rate was notably low, where among the treated, only 53.2% had their BP controlled and only 36.4% of all those with HTN, leaving close to two thirds of patients at greater cardiovascular risk. Prevalence of HTN was higher in women and increased with age. Initially, differences in prevalence were found among categories of other sociodemographic variables; however, once adjusted for age, no differences were found. HTN awareness and treatment were both higher in women and in older participants, and being aware was the main determinant for treatment. Overall control was higher in women and older age groups; however, in those with treatment for HTN, there was no significant difference in control rates between sex or age groups. These findings are similar to studies done in other cities of South America, where the majority of the hypertensive population has awareness, roughly half are on treatment, but only a minority are controlled.^12^, ^13^ In contrast, in places like China and Korea, studies have shown much lower awareness, treatment, and control, each parameter estimated to be present in less than 50% of the population.^15^, ^16^

The mean SBP was 120.3 mm Hg, and the mean DBP was 78.2 mm Hg. Men had an overall higher SBP than women. The difference in SBP between men and women is higher in younger age groups (49 years or younger) and decreases to similar levels at older ages (70 years or older). Both SBP and DBP increase, on average, with age; however, the increase in SBP is linear, and DBP stops increasing between 50 and 60 years. These figures are near the mean SBP and DBP reported in other Latin American studies like those done in Santander (Colombia), the CARMELA study, and CAMDI Nicaragua, which reported BP of (SBP/DBP mm Hg) 116.4/74.1,^26^ 116.1/75.8,^12^ and 123.2/75.5,^25^ respectively, showing also a similar distribution by sex and age.

In other studies, the prevalence of HTN has been shown to have a linear association with age; however, varying results have been reported regarding sex, where some have found a higher prevalence in men (Argentina),^27^ others have found it in women (Costa Rica),^28^ and some report no difference at all (Central America and Mexico)^7^, ^29^; however, most of the revised literature shows men as having, in general, higher prevalence of HTN than women.^12^, ^13^, ^14^ The CAMDI survey, which estimated the prevalence of HTN in all Central American capital cities, found that only in Managua did women have a significantly higher prevalence than men,^7^ which is consistent with our findings and other research done in Nicaragua.^30^, ^31^ Additional unpublished data from our study regarding the prevalence of risk factors for chronic diseases suggest that the higher prevalence of HTN in women in our population may be explained by the higher prevalence of obesity and other metabolic risk factors in this group than in men. It is possible that cultural differences may be an important underlying factor that determines these differences between men and women in different regions of the world. For instance, traditional gender roles are seen in this population, where men and women commonly engage in different activities and lines of work. Men in San Rafael del Norte are generally tasked with physical work with a higher metabolic demand than women, who generally work in the home setting or have sedentary jobs. These data will be published separately.

An important association is suggested between awareness, treatment, and control, as it can be seen when comparing these rates by sex, where women have higher rates of all three indicators than men. This finding is consistent with studies across all regions of the world, where women have notably higher awareness, treatment, and control than men. Furthermore, the observed difference between the treatment and control rates by sex and all other groups is consistently near 30%. However, when considering those on treatment only, still almost half do not achieve adequate BP control, and this proportion does not significantly change by sex, age, and other variables. Even though the predictors for controlled HTN are still to be determined by future research, we can hypothesize that inadequate control could be related to inadequate medical treatment, such as pharmacological nonadherence, ineffective prescription, low‐quality medication, among others, and prevalent lifestyle risk factors. In regard to age groups, the differences in treatment and control rates are also associated with awareness, where younger age groups had significantly less awareness than older age groups; however, when considering only those with treatment, the control rate was also close to 50%, and there were no differences between groups.

### 4.1 Strengths and limitations

The Elieth‐HIFARI study is the first in Nicaragua to use the recommendations of the WHL for the standardized reporting of the prevalence of HTN, awareness, treatment, and control, as well as Pre‐HTN and optimal BP. These indicators and unified criteria will facilitate comparisons with trends in future research. This is also the first study to implement the protocol of the WHO's STEPwise method for risk factor surveillance. Even though our results cannot be extrapolated to other populations of Nicaragua, they are representative of a typical small town of the northern region of the country, as it possesses important shared health determinants such as agricultural activities, eating habits, less agitated lifestyles, and cooler climate, constituting the most important local evidence thus far reported. Additionally, this is the first study to employ a population‐based survey using a random stratified sampling method that provides a much more reliable representation of the population and more accurate estimate of prevalence rates, in contrast with many other studies done in Nicaragua, which have been done using convenience sampling.^30^, ^31^

Nevertheless, there were various limitations in this study. The most relevant limitation was the fact that the new diagnosis of HTN was based on only one visit without subsequent visits to confirm, which was logistically unfeasible. Most population‐based surveys, however, have followed a similar design. Because of this, there is a risk of overestimating the prevalence of HTN. The sample size of our study was also a limitation for determining statistical differences between subgroups of smaller quantities, such as those older than 70 years, subgroups of age and sex, and subgroups of participants with HTN, awareness, and treatment. In these instances, the assumptions for normality and other requirements for statistical testing were not satisfied for analysis.

### 4.2 Implications and future research

In order to achieve a substantial reduction in HTN and the cardiovascular complications that can arise from it, it is essential to adequately surveil the prevalence of HTN to determine its importance as a public health priority and the effectiveness of policy measures designed to reduce it. Consequently, our study provides the starting point for these purposes in a typical small town of northern Nicaragua.

Awareness is a key aspect in avoiding cardiovascular complications as it is associated to higher rates of treatment and control. The WHL states: “population‐level awareness of hypertension indicates the efficiency of community and clinical resources in identifying people who might benefit from clinical management of hypertension.”^10^ Our results indicate the need for systematic screening and case‐finding interventions for the general population, targeted especially at men and younger individuals, groups that have lower awareness rates. As admitted by the WHL, it is not reasonable to assume that all who have HTN require pharmacotherapy, as there are varying criteria and some may be controlled with only lifestyle changes. We found a high rate of treatment among those who were aware of having HTN; therefore, access and availability to health care interventions does not seem to be a major problem in this population. However, despite the high rate of treatment among those aware, the majority of those with HTN were not controlled; therefore, there is much room for improvement in the health system's efforts of identifying and effectively treating hypertensive patients.

Further research must be conducted to explain the significantly higher prevalence of HTN that women have compared with men in Nicaragua. It is important to determine the causes of low BP control in the hypertensive population and especially in those who have treatment. Periodic estimates of HTN prevalence, awareness, treatment, and control must be conducted to evaluate progress.

## 5 CONCLUSIONS

The prevalence of HTN in San Rafael del Norte is high, encompassing more than a quarter of the adult population, and constitutes a major public health problem. The high prevalence of HTN increases the risk for cardiovascular complications, especially in older adults and women. Awareness is a key determinant for treatment and control, and efforts should be made to increase awareness among the population, especially in men and younger adults. However, despite having awareness and treatment, adequate control is still lacking in a significant fraction of both sexes. Future research should be conducted to evaluate the progress in reducing HTN prevalence and increasing awareness and control. Also, further research should aim to clarify the disparity of HTN prevalence between men and women in Nicaragua.

## FUNDING

Self‐funded.

## CONFLICTS OF INTEREST

The authors have nothing to disclose.

## AUTHOR CONTRIBUTIONS

Conceptualization: Marion Jose Valladares, Noel Rodriguez, Ginner Rizo, Indiana Lopez

Data Curation: Marion Jose Valladares, Noel Rodriguez

Formal Analysis: Marion Jose Valladares, Noel Rodriguez, Indiana Lopez

Funding Acquisition: Ginner Rizo, Marion Jose Valladares, Noel Rodriguez

Investigation: Noel Rodriguez, Marion Jose Valladares, Marco Rodriguez, Rosse Rivera

Methodology: Marion Jose Valladares, Noel Rodriguez, Indiana Lopez

Project Administration: Marion Jose Valladares, Noel Rodriguez

Resources: Ginner Rizo, Marion Jose Valladares, Noel Rodriguez

Supervision: Marion Jose Valladares, Noel Rodriguez, Ginner Rizo, Indiana Lopez

Writing – Original draft: Marion Jose Valladares

Writing – Review & Editing: Marion Jose Valladares, Noel Rodriguez, Ginner Rizo, Marco Rodriguez, Rosse Rivera, Indiana Lopez

All authors have read and approved the final version of the manuscript.

Marion Jose Valladares and Noel Rodriguez had full access to all of the data in this study and take complete responsibility for the integrity of the data and the accuracy of the data analysis.

## TRANSPARENCY STATEMENT

Marion Jose Valladares affirms that this manuscript is an honest, accurate, and transparent account of the study being reported; that no important aspects of the study have been omitted; and that any discrepancies from the study as planned (and, if relevant, registered) have been explained.
